# Inductions of granulosa cell luteinization and cumulus expansion are dependent on the fibronectin-integrin pathway during ovulation process in mice

**DOI:** 10.1371/journal.pone.0192458

**Published:** 2018-02-08

**Authors:** Hiroya Kitasaka, Tomoko Kawai, S. A. Masudul Hoque, Takashi Umehara, Youko Fujita, Masayuki Shimada

**Affiliations:** 1 Laboratory of Reproductive Endocrinology, Graduate School of Biosphere Science, Hiroshima University, Higashi-Hiroshima, Hiroshima, Japan; 2 Asada Ladies Clinic, Nagoya, Japan; 3 Bangabandhu Sheikh Mujibur Rahman Agricultural University, Gazipur-1706, Bangladesh; 4 Women’s Clinic Oizumi-Gakuenn, Higashi-Oizumi, Tokyo, Japan; Universite Clermont Auvergne, FRANCE

## Abstract

It has been known that EGF-like factor secreted from LH-stimulated granuloma cells acts on granulosa cells and cumulus cells to induce ovulation process. Granulosa cells are changed the morphology with differentiating cell functions to produce progesterone. Cumulus cells are detached to make a space between the cells to accumulate hyaluronan rich matrix. LH also changes extracellular matrix (ECM) components including fibronectin in the follicular walls and granulosa cell layers. EGF like factor and fibronectin synergistically play important roles in numerous cell functions, especially cancer cell migration, estimating that fibronectin would impact on granulosa cells and cumulus cells. To clear this hypothesis, the localizations of fibronectin and its receptor integrin were observed by immunofluorescence technique. The functions were monitored by the detection of downstream signaling pathway, focal adhesion kinase (FAK). The pharmacological approach in both *in vivo* and *in vitro* were used for analyzing the physiological roles of FAK during ovulation process. The immunofluorescence staining revealed that fibronectin and integrin were observed in granulosa cells, cumulus cells and the space between cumulus cells and oocyte at 4 and 8 h after hCG injection. Concomitantly with the changes of fibronectin-integrin localization, FAK was phosphorylated in periovulatory follicles. The injection of FAK inhibitor suppressed not only ovulation but also luteinization of granulosa cells and cumulus expansion. In cultured-granulosa cells, fibronectin-integrin synergistically activated FAK with amphiregulin (AREG). Such cooperative stimulations induced a morphological change in granulosa cells, which resulted in the maximum level of progesterone production via the induction of *Hsd3b*. When cumulus-oocyte complexes (COCs) were cultured with AREG in the presence of serum, the maximum level of cumulus expansion was observed. The AREG-induced cumulus expansion was also suppressed by FAK inhibitor. Thus, it is concluded that fibronectin and AREG synergistically activate FAK not only in granulosa cells and cumulus cells to induce successful ovulation process.

## Introduction

Luteinizing hormone (LH) is transiently secreted from pituitary glands and acts on its receptor expressed on granulosa cells to induce ovulation process [1]. In LH-stimulated granulosa cells, epidermal growth factor (EGF)-like factor is expressed and secreted to act on its receptor, EGFR, in both granulosa cells and cumulus cells [2][3]. The downstream pathway of EGFR in follicular somatic cells is mitogen-activated protein kinase (MAPK1/3), also called ERK1/2, because in *Mapk1*^*-/-*^*;Mapk3*^*flox;flox*^*;Cyp19Cre* mice that MAPK1/3 are deleted in granulosa cells and cumulus cells, ovulation is completely suppressed [4]. In the mutant mouse, almost all genes reported to be expressed in granulosa cells and cumulus cells during ovulation process are not expressed after human chorionic gonadotropin (hCG) injection [4], indicating that the function of EGFR changes the gene expression pattern from the follicular development stage to ovulation process.

Activation of EGFR is also involved in cell migration and morphological changes of the cell shape [5]. The EGF-like factor-EGFR pathway increases the enzymatic activity of calpain 2 via both Ca^2+^ induction and ERK1/2 activation in cumulus cells during ovulation process [6]. Calpain-degraded focal adhesion components, such as paxicillin and tallin, induce cytoskeleton remodeling in cumulus cells, and this induction is essential for the detachment of cumulus cells and cell migration during ovulation process of the mouse cumulus-oocyte complex (COC) [6]. Modification of the cytoskeleton is also observed in granulosa cells during ovulation process [7][8]. When the mutant type of cofilin, one of the focal adhesion mediators, was overexpressed in cultured granulosa cells, not only actin remodeling but also progesterone production was not induced in the cells [9]. Cofilin is known to be regulated by an EGFR-dependent pathway in fibroblasts [10], indicating that the EGF-like factor-EGFR pathway induces differentiation of granulosa cells and cumulus cells during ovulation process via both changes in gene expression patterns and morphological changes of the cells. However, the latter mechanisms that change the shape of both granulosa cells and cumulus cells have been unclear.

Focal adhesion components bind to integrins, which are transmembrane proteins, and recognize changes in extracellular environments to maintain or change the cell shape [11][12]. The major binding partner of integrins is fibronectin, a component of the extracellular matrix (ECM) of organs including ovarian follicles [13]. During follicular development and ovulation, ECM components change kinetically in the follicular walls and granulosa cell layers. In preantral and early antral follicles, the follicular walls consist of laminin, collagen and perlecan [14]. After the LH surge, granulosa cells express fibronectin and laminin that accumulate within the granulosa cell layers [15]. These changes in ECM components affect granulosa cell functions at least *in vitro*. Huet et al. [16] reported that, when granulosa cells were cultured on a collagen-coated surface, follicle-stimulating hormone (FSH) enhanced estrogen production compared with granulosa cells without collagen. Conversely, coating with fibronectin or laminin induces progesterone production, suggesting that ECM components support EGF-like factor-EGFR-induced luteinization of granulosa cells. Focal adhesion kinase (FAK) is an assembly point of both signaling pathways, where integrins mediate the phosphorylation of Tyr397 of FAK and the EGFR pathway induces phosphorylation of Tyr925 of FAK in a Src-dependent manner [17][18]. The both phosphorylation sites of FAK leads to the maximum enzymatic activity of FAK to induce the differentiation and migration of cells [19]. Therefore, we hypothesized that EGFR and integrin signaling pathways, which change the shapes of granulosa cells and cumulus cells, would be essential for the differentiation of both cell types. However, there is limited information about the relationship between the functions of integrins and the EGFR pathway in cumulus cells and granulosa cells during ovulation. Moreover, little is known about the direct or indirect effects of ECM components in periovulatory follicles on oocyte maturation.

To test the above hypothesis, we focused on fibronectin in granulosa cells, cumulus cells and oocyte during ovulation process. We investigated the kinetic changes of fibronectin and integrin in periovulatory follicles and their roles in activation of the FAK pathway. Using primary *in vitro* culture of granulosa cells, we also examined whether fibronectin and/or EGF are required for the phosphorylation of FAK. Finally, using a pharmacological approach, the roles of FAK and the binding of fibronectin to integrins in granulosa cells and cumulus cells during ovulation were analyzed *in vivo* and *in vitro*.

## Materials and methods

### Materials

Equine chorionic gonadotropin (eCG) and hCG were purchased from Asuka Seiyaku (Tokyo, Japan). Dulbecco’s modified Eagle’s medium (DMEM)-F12 medium and penicillin-streptomycin from Invitrogen (Carlsbad, CA, USA); oligonucleotide poly-(dT) from Invitrogen, AMV reverse transcriptase from Promega (Madison, WI, USA). Routine chemicals and reagents were obtained from Nacalai Chemical Co (Osaka, Japan) or Sigma (St. Louis, MO, USA).

### Animals

Immature female C57BL/6 mice (3 weeks old) were obtained from Charles River Japan (Yokohama, Japan). At 23 days of age, female mice were injected intraperitoneally (i.p.) with 4 IU eCG to stimulate follicular growth, followed by 5 IU hCG after 48 h to stimulate ovulation and luteinization. Animals were housed under a 12 h light/12 h dark cycle in the Experiment Animal Center at Hiroshima University and provided with food and water ad libitum. Mice were euthanized using CO_2_ administration method. The animals were treated in accordance with the NIH Guide for the Care and Use of Laboratory Animals. The experimental design was approved by the Animal Care and Use Committee at Hiroshima University (ID number C-13-18).

### Treatment of mice with FAK inhibitor Y15

Immature female mice were injected i.p. with 4 IU eCG. After 48 h, the mice were injected i.p. with 5 IU hCG and/or 30 mg/kg Y15 (Tocris Bioscience, Bristol, UK) [20]. The treatment significantly suppressed the phosphorylation of FAK (Tyr925) in ovaries at 4 h after hCG injection when the maximum level of phosphorylation was induced by hCG (S1 Fig). At 4, 8, 10, and 16 h after hCG injection, their ovaries were collected. Ovaries collected from three mice in each treatment group at each time point (0, 8, 10 or 16 h after hCG injection) were stained with hematoxylin-eosin (Sakura-Finet Japan, Osaka, Japan). RNA was purified from granulosa cells isolated from periovulatory follicles of ovaries of one mouse and then used for real-time PCR. Three mice in each treatment group at each time point (0, 4 or 8 h after hCG injection) were used for RNA analyses. At 16 h after hCG injection, we collected the ovulated COCs from oviducts of five mice in each treatment group and counted the number of ovulated oocytes.

### Real-time PCR

Total RNA was obtained from mouse ovaries using the RNAeasy Mini Kit (Qiagen Sciences, Germantown, MD, USA) according to the manufacturer’s instructions, and real-time PCR analyses were performed as described previously [21]. Briefly, total RNA was reverse transcribed using 500 ng poly-dT (Invitrogen) and 0.25 U avian myeloblastosis virus-reverse transcriptase (Promega Corp., Madison, WI, USA) at 42°C for 75 min and then 95°C for 5 min. cDNA and primers were added to a 15 μl total reaction volume of Power SYBR Green PCR Master Mix (Applied Biosystems, Foster City, CA, USA). PCRs were then performed using the StepOne real-time PCR system (Applied Biosystems). Conditions were set to the following parameters: 10 min at 95°C followed by 45 cycles of 15 sec at 95°C and 1 min at 60°C. Specific primers pairs were selected and analyzed as indicated in S1 Table.

### Western blot analyses

Protein samples from ovaries and granulosa cells were prepared by homogenization in cell lysis buffer (cOmplete™ Lysis-M EDTA-free, Roche) and then diluted in the same volume of Sample Buffer Solution with 2-ME (2x) for SDS-PAGE (Nacalai Chemical Co). Extracts (30 μg protein) or precipitates were resolved by SDS polyacrylamide gel (7.5%) electrophoresis and transferred to polyvinylidene fluoride membranes (GE Healthcare Life Sciences, PA, USA). The membranes were blocked in Tris-buffered saline and Tween 20 (TBST, 10 mM Tris, pH 7.5, 150 mM NaCl, and 0.05% Tween 20) containing 5% (w/v) nonfat carnation instant milk (Nestle Co., Solon, OH, USA) or 5% (w/v) bovine serum albumin (BSA, Nacalai Chemical Co). Blots were incubated with a primary antibody (S2 Table) overnight at 4°C. After washing in TBST, enhanced chemiluminescence (ECL) detection was performed using an ECL system (GE Bioscience) according the manufacturer’s specifications, followed by appropriate exposure of the blots to Fuji X-ray film (Fujifilm, Tokyo, Japan).

### Immunofluorescence staining

Whole ovaries collected from immature mice treated with or without eCG were fixed in 4% (w/v) paraformaldehyde (Nacalai Chemical Co) for 24 h at 4°C. Subsequently, these tissues were washed with PBS and embedded in paraffin after dehydration. Paraffin-embedded tissue sections (5 μm thick) were deparaffinized and treated with 30% (v/v) H_2_O_2_ in methanol to block endogenous peroxidases. After washing with PBS, the sections were boiled for 20 min in 10% citric acid (pH 6.0) for antigen activation. Then, the sections were washed with PBS and blocked using 5% (w/v) BSA in PBS or MOM Immunodetection Kit (Vector Laboratories Inc, CA, USA). The sections were incubated with primary antibodies (S2 Table) in 1% (v/v) blocking solution overnight at 4°C. After washing with 0.3% (v/v) Triton X-100 in PBS, the staining was visualized with Cy3-conjugated goat anti-rabbit IgG (1:200) (Sigma) and FITC-conjugated goat anti-rabbit IgG (1:200) with counterstaining by 4,6-diamidino-2-phenylindole (DAPI, Vector Laboratories Inc).

Cultured granulosa cells and oocytes were fixed in 4% (w/v) paraformaldehyde for 30 min at room temperature. Subsequently, these cells were washed with PBS and treated with PBS containing 0.3% (v/v) Triton X-100 (Sigma). Then, these cells were washed with PBS and incubated with rhodamine-phalloidin (1:200) (Invitrogen) and DAPI. Oocytes were also treated with α/β Tubulin and visualized with FITC-conjugated goat anti-rabbit IgG (1:200). Digital images were captured using a BZ-9000 microscope (Keyence Co., Osaka, Japan).

### Hematoxylin-eosin staining

Ovaries were fixed and then embedded in paraffin. After paraffin-embedded tissue sections (5 μm thick) were deparaffinized and washed with PBS, the sections were stained with hematoxylin-eosin to visualize the nuclei and cytoplasm, respectively. Tissues were observed under a light microscope.

### *In vitro* culture of granulosa cells

Granulosa cells were harvested by needle puncture from immature mice treated with eCG at 23 days of age. Briefly, 1×10^6^ cells were cultured in a 24-well fibronectin-coated plate (human fibronectin cell ware, BioCoat, MA, USA) or uncoated 24-well culture plate (142485, Nunc) in serum-free medium (DMEM:F12 medium containing penicillin-streptomycin) with a lipid mixture (L-2088, Sigma) and 3 μg/ml fibronectin (F-1141, Sigma) with or without 100 ng/ml amphiregulin (AREG, R&D Systems, Minneapolis, MN, USA) and either 100 nM FAK inhibitor (Y15) that suppressed the phosphorylation of FAK at Tyr925 (S1 Fig) or 10 μM EGFR inhibitor (AG1478, BIOMOL). Because the culture medium did not contain serum or follicular fluid, and because progesterone was produced from cholesterol that was mainly taken to granulosa cells but was slightly *de-novo* synthesized in granulosa cells, lipid mixture containing cholesterol was added as a source of steroidogenesis. Because follicular fluids contain fibronectin, fibronectin was also added to serum-free medium.

After 24 h, granulosa cells were collected for western blot or real-time PCR analyses, and the culture supernatant was collected for measurement of progesterone. For immunofluorescence staining, granulosa cells were cultured on glass plate. Because it has been known that serum contains fibronecin, granulosa cells were cultured in fetal bovine serum (FBS, cat#10438–018, Gibco)-coated or uncoated glass bottom dishes in serum-free medium (DMEM:F12 containing penicillin-streptomycin) with 100 ng/ml AREG and 3 μg/ml fibronectin for 12 h.

### Isolation and *in vitro* maturation of COCs

Ovaries of immature mice primed with eCG for 48 h contain multiple preovulatory follicles. COCs were isolated from these follicles by needle puncture and collected by a pipette. Non-expanded COCs were selected, and groups of 50 COCs were cultured in a 50 μl drop of defined medium containing 1% (v/v) of FBS (Sigma) with 100 ng/ml AREG and/or either 100 nM FAK inhibitor (Y15) or 100 μM cyclo RGD peptide (Sigma) in a 35-mm dish in the presence of 4 mM hypoxanthine (Sigma). FBS was added to this culture medium because FBS-containing inter α trypsin inhibitor has been known to be essential for cumulus expansion. The dose of RGD peptide has been reported to suppress the binding of fibronectin to integrins [22]. The area of each expanded COC was measured using the BZ-9000 microscope.

### Quantitative determination of progesterone levels

The progesterone levels in serum and conditioned medium of granulosa cells were determined using a rodent progesterone ELISA test kit (Endocrine Technologies, Inc., San Francisco, USA) according to the manufacturer’s instructions. Medium samples (50 μl each) were analyzed using a microplate reader to determine the amount of converted substrate at 450 nm.

### Measurements of the surface area of an expanded COC, the size of cultured granulosa cells, and the number of stress fibers in cultured granulosa cells

To determine the area of each expanded COC in ovarian sections, the sections were stained with hematoxylin-eosin. The surface area of each COC was assessed in sections containing an oocyte with a nucleus. An area measurement system associated with the BZ-9000 microscope was used for measurements of the surface area of expanded COCs. Fifteen COCs were analyzed in each ovary. The ovaries were collected from three mice in each treatment group at each time point.

To determine the surface area and number of stress fibers in the cultured granulosa cells, they were stained for F-actin. For quantitative analysis of the area or the number of stress fibers in each granulosa cell, Z-stacks were captured at seven steps over a Z-axis distance of 0.3 μm using the BZ-9000 microscope. Stacks were reconstructed and analyzed by the Keyence application for the BZ-9000, which allowed us to determine the fluorescent area and count the number of stress fibers in each cell. Fifteen cells per culture were analyzed.

### Statistics

Statistical analyses of data from three or four replicates for comparison were carried out by Student's t-test, one-way analysis of variance (ANOVA) or two-way ANOVA followed by Student's t-test (Statview, Abacus Concepts, Inc., Berkeley, CA).

## Results

**Fig 1:**
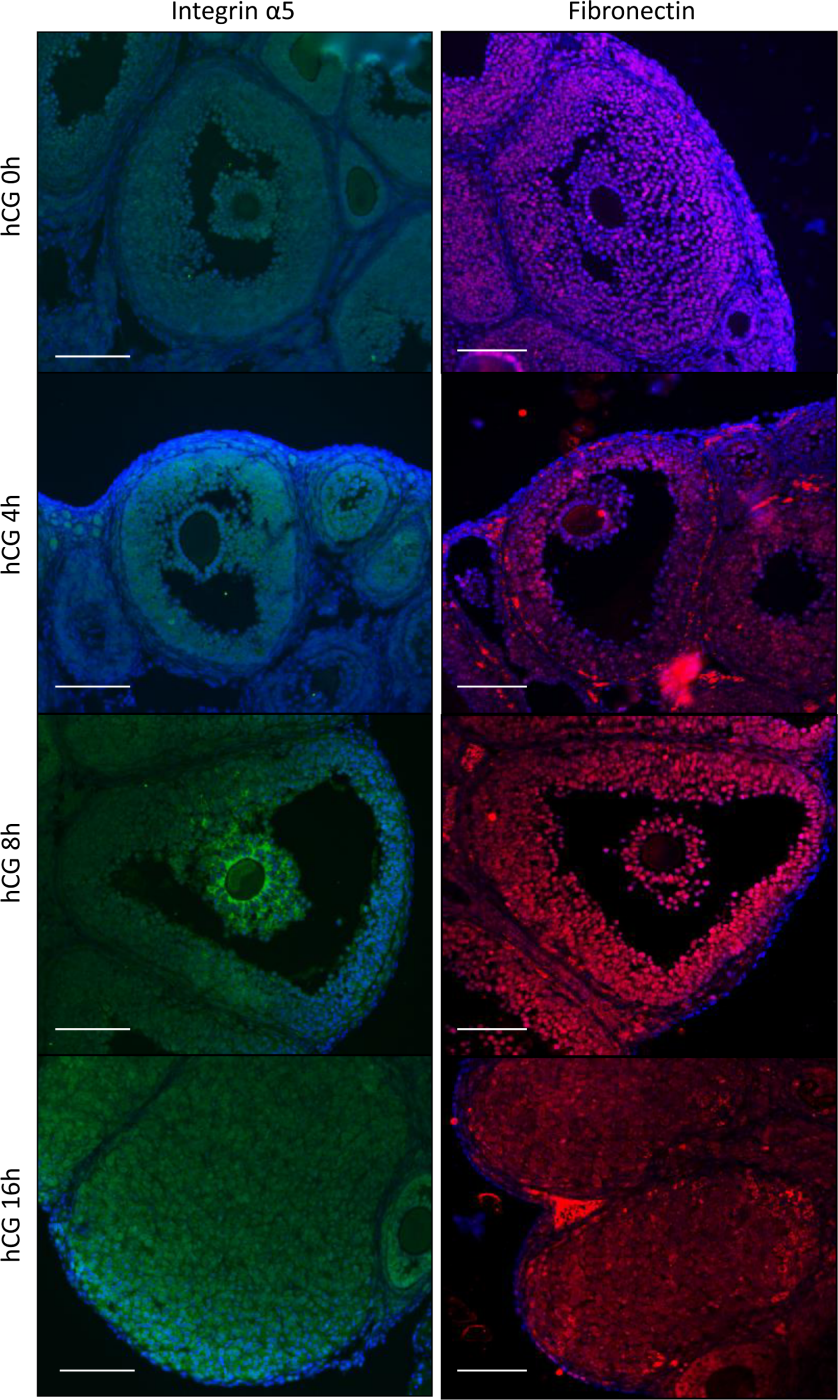
Expression of fibronectin and integrins in the mouse ovary during ovulation. Localization of fibronectin and integrin α5 in the mouse ovary was detected by immunofluorescence staining (n = 3 mice in each time point). Proteins were visualized with Cy3 (fibronectin) and FITC (integrin α5). The nucleus was counterstained with DAPI. Scale bar is 100 μm.

Their localization of fibronectin and integrin α5 was analyzed by immunofluorescence staining. Positive signals for integrin α5 were detected in granulosa and cumulus cells in preovulatory and periovulatory follicles (Fig 1). Strong signals of integrin α5 were observed in the inner layers of cumulus cells at 8 h after hCG injection (Fig 1). Fibronectin was observed on granulosa and cumulus cells of preovulatory follicles. The signals were also detected after hCG injection and the positive signals for fibronectin were also observed in the corpus luteum (Fig 1).

### Kinetic changes of the phosphorylation status of FAK in the mouse ovary during ovulation

**Fig 2:**
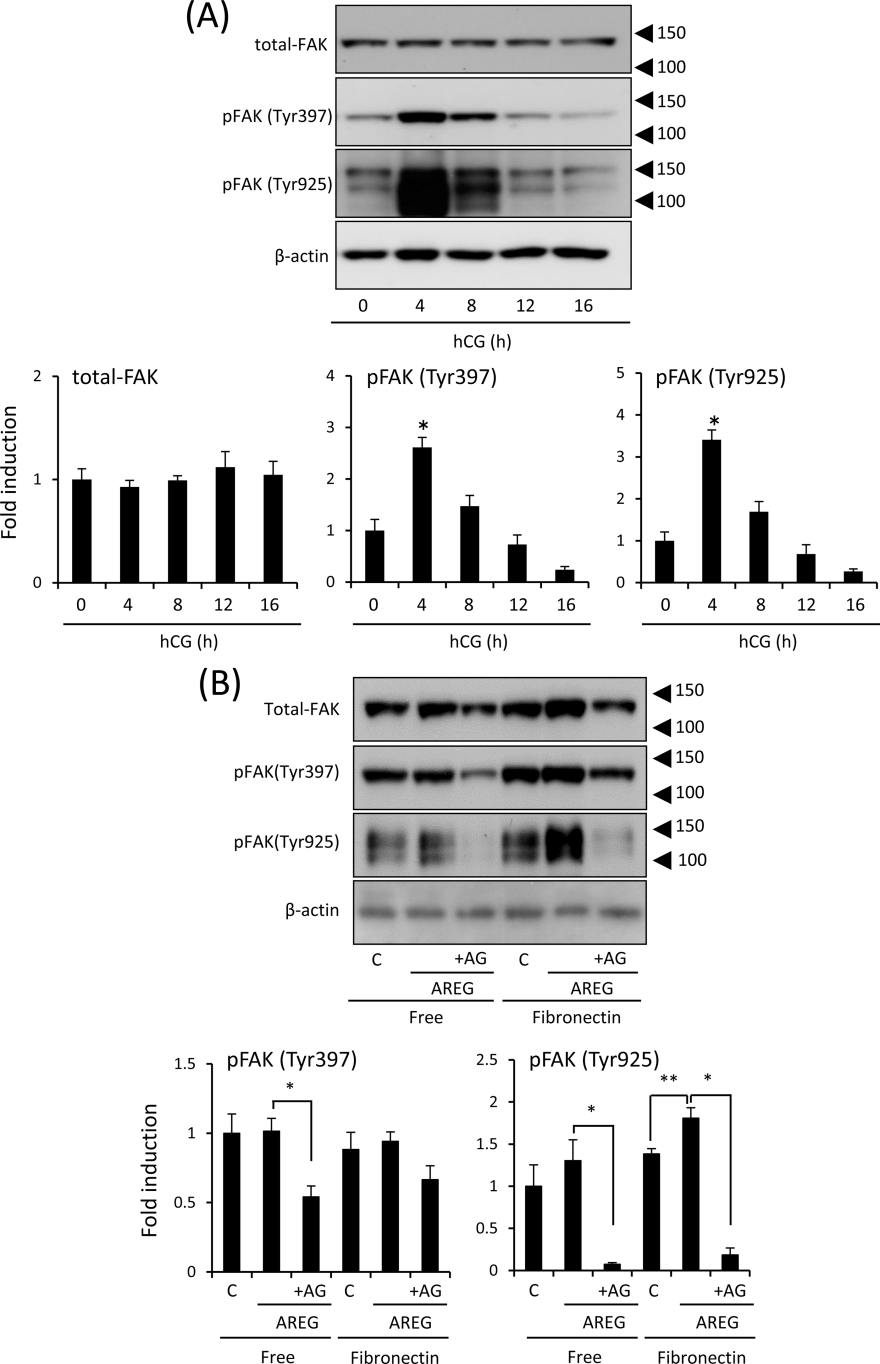
Expression of phosphorylation of FAK in the mouse ovary during ovulation. (A): Levels of FAK (total FAK), phosphorylated forms of FAK (Tyr397 and Tyr925), and β-actin in whole ovary samples were detected by western blot analyses. The ovary was collected from mice treated with hCG. Three mice were used for ovarian sampling at each time point. β-actin was used as a loading control. The intensity of the bands was analyzed and values are mean +/- SEM of 3 replicates. *; Significant differences were observed by hCG injection as compared with those before hCG injection (0h) (P<0.05). (B): Phosphorylation status of FAK in granulosa cells cultured for 4 h with or without AREG on uncoated or fibronectin-coated plates. C (control): granulosa cells cultured without AREG; AREG: granulosa cells treated with 100 ng/ml AREG; AG: granulosa cells treated with both AREG and 10 μM AG1478 (AG, a selective EGFR tyrosine kinase inhibitor); Free: granulosa cells cultured on uncoated wells; Fibronectin: granulosa cells cultured on fibronectin-coated wells. β-actin was used as a loading control. The intensity of the bands was analyzed and values are mean +/- SEM of 3 replicates. *; Significant differences were observed by the addition of AG1478 (AG) (P<0.05). **; The treatment with AREG significantly increased the intensity of pFAK (Tyr925) in granulosa cells cultured on the fibronectin-coated wells (P<0.05).

The total amount of FAK was not dramatically changed in the ovary during ovulation (Fig 2A). The single positive signal of the anti-phospho-Tyr397 FAK antibody was detected at 125 kDa (Fig 2A). The intensity was significantly increased at 4 h after hCG injection. Two bands were detected by the anti-phospho-Tyr925 FAK antibody at 125 and 150 kDa (Fig 2A). The 125 kDa band was positive and the 150 kDa was non-specific because total FAK and Tyr397-phosphorylated FAK were detected at 125 kDa. The intensity of the 125 kDa band of the anti-Tyr925 FAK antibody was also significantly increased at 4 h after hCG injection. At 12 h after hCG injection, both phosphorylated FAK signals had decreased to the basal level before hCG injection (Fig 2A).

To clarify the relationship between fibronectin and phosphorylation of FAK in granulosa cells, the cells were cultured on a fibronectin-coated plate. Phosphorylation of Tyr397 was not induced by fibronectin-coated plate and by additional AREG to non-coded plate (Fig 2B). The phosphorylation of Tyr925 was not induced by either fibronectin or AREG alone as similar to Tyr397. However, both sites were phosphorylated by AREG when granulosa cells were cultured on the fibronectin-coated pate (Fig 2B).

### Effects of the FAK inhibitor on ovulation including cumulus expansion of COCs, luteinization of granulosa cells, and release of oocytes to the oviduct

To clarify the roles of FAK activated by both fibronectin and EGF-like factor in granulosa cells during ovulation, FAK inhibitor Y15 was co-injected with hCG, and then we examined its effects on ovulation, cumulus expansion of COCs, and both the morphology and function of granulosa cells.

**Fig 3:**
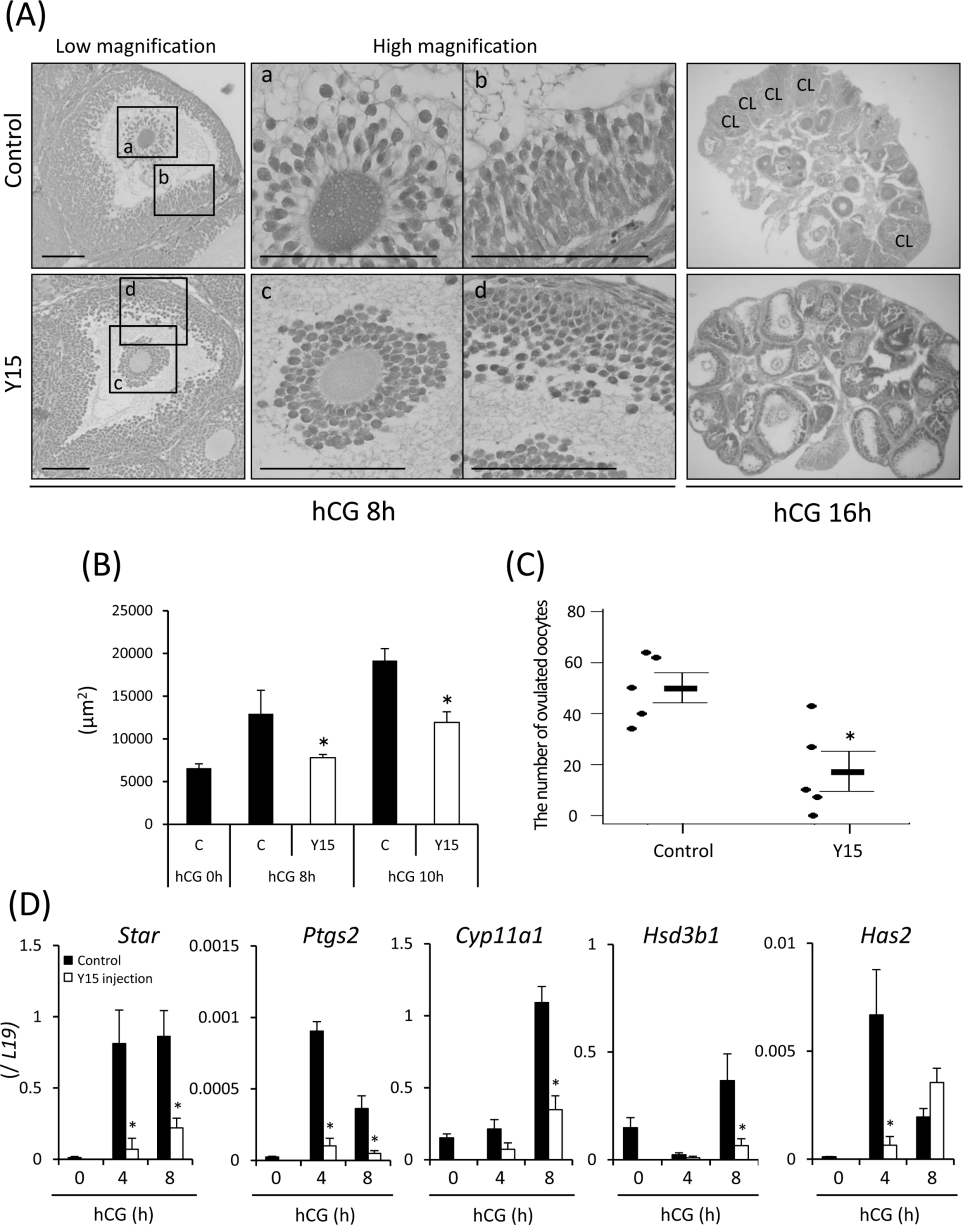
Effects of FAK inhibitor Y15 during ovulation *in vivo*. (A): Ovarian morphology of mice treated with hCG for 8 or 16 h was observed by hematoxylin-eosin staining (n = 3 ovaries in each group at each time point). Y15: eCG-primed mice co-injected with hCG and Y15 (30 mg/kg). Scale bar is 100 μm. (B): Effect of Y15 on cumulus expansion (surface area of COCs in periovulatory follicles of the ovary). Ovaries were collected at 0, 8, and 10 h after hCG injection with or without Y15 (30 mg/kg). The ovarian tissue was stained with hematoxylin-eosin, and individual areas of COCs were measured in periovulatory follicles in sections containing an oocyte with a nucleus. C (control): eCG-primed mice injected with hCG; Y15: eCG-primed mice co-injected with hCG and Y15 (30 mg/kg). *; Treatment with Y15 significantly decreased the mean surface area of COCs compared with the control at each time point (p<0.05). Values are the mean ± SEM of five COCs each in three sections. (C): The number of ovulated oocytes when mice were co-administered with hCG and Y15. Ovulated oocytes were collected from oviducts and counted at 16 h after hCG injection. Values are the mean ± SEM of five mice. *; Significant differences were observed compared with the control (p<0.05). C (control): Immature female mice treated with eCG followed by hCG stimulation; Y15: mice co-injected with hCG and Y15 (30 mg/kg) at 48 h after eCG injection. (D): Expression of genes involved in progesterone production and hyaluronic acid synthesis in the ovary of mice treated with hCG and Y15 (30 mg/kg). Three mice were used for sampling granulosa cells in each treatment group at each time point. The mRNA levels of *Star*, *Cyp11a1*, *Hsd3b1*, *Ptgs2* and *Has2* were analyzed by real-time PCR and normalized to that of *L19*. Values are the mean ± SEM of three replicates. *; Y15 treatment significantly suppressed the expression of each gene compared with the control at each time point (p<0.05).

At 8 h after hCG injection, cumulus cells had detached from the outer layers (Fig 3A) and the area of COCs was increased significantly in the control ovary (Fig 3B). However, in mice injected with both hCG and Y15, the cumulus layers were tightly surrounding oocytes and the area of COCs was significantly smaller than in the control (Fig 3A and 3B). At high magnification, both cumulus cells and granulosa cells had changed to an elliptical shape in control mice (Fig 3A). However, treatment with Y15 did not induce the morphological change of cumulus cells and granulosa cells, and the cells remained in a spherical shape (Fig 3A). At 16 h after hCG injection, ovulation occurred and the corpus luteum was formed in the ovary (Fig 3A). There were more than 50 ovulated oocytes in control mice (Fig 3C). However, oocytes with cumulus cell layers were still observed in most antral follicles when mice were treated with hCG and Y15 (Fig 3A). The number of ovulated oocytes was significantly lower in the hCG and Y15 co-treatment group compared with the control (Fig 3C). The expression of genes involved in luteinization (*Star*, *Cyp11a1*, and *Hsd3b1*) of granulosa cells and cumulus expansion (*Has2*) of cumulus cells was induced by hCG injection (Fig 3D). However, co-injection with Y15 significantly inhibited their inductions (Fig 3D).

### Fibronectin and EGF-like factor synergistically induce both the morphological change and the progesterone production of granulosa cells

**Fig 4:**
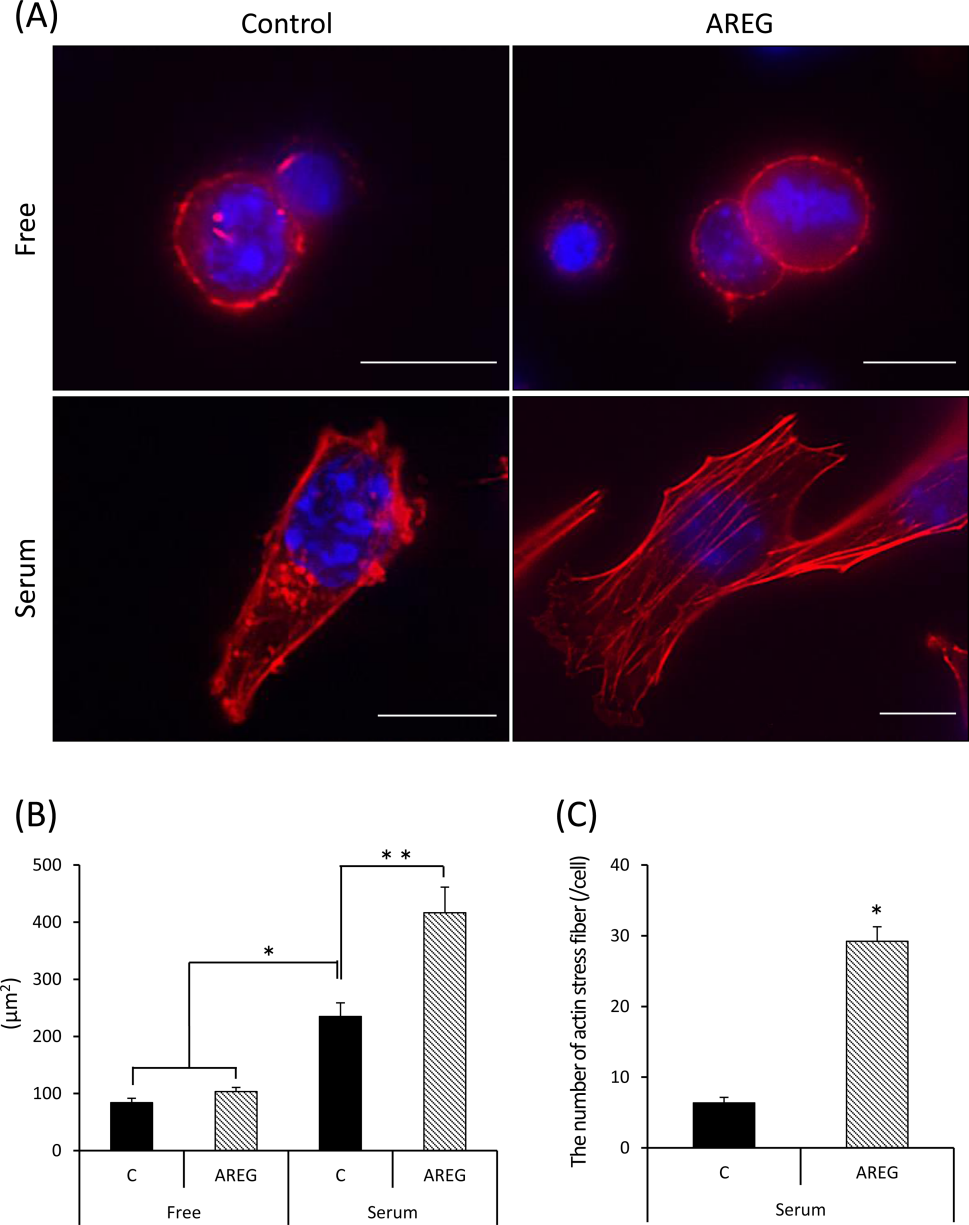
Synergistic effects of fibronectin/serum coating and AREG stimulation on the morphology of granulosa cells *in vitro*. (A): Granulosa cells were collected from the ovary of mice treated with eCG for 48 h and cultured in uncoated wells (upper panel, Free) or serum-coated wells (lower panel, serum) with (right panel, AREG) or without (left panel, control) AREG. After 24 h, the cultured granulosa cells were stained for F-actin and counterstained with DAPI. Scale bar is 10 μm. (B): Surface area of granulosa cells cultured for 24 h with or without AREG on uncoated or serum-coated wells. The surface area of each granulosa cell was measured by an area measurement system associated with the BZ-9000 microscope. Values are the mean ± SEM of fifteen granulosa cells in each treatment group of three independent experiments (45 granulosa cells in total were measured in each treatment group). Data were statistically analyzed by two-way ANOVA. Because the significant difference was observed for each factor, the comparison analysis was performed by the Student’s t-test. *; AREG significantly increased the area of granulosa cells cultured on serum-coated wells (p<0.05). Free: granulosa cells cultured on uncoated wells; serum: granulosa cells cultured on serum-coated wells; C (control): granulosa cells treated without AREG; AREG: granulosa cells treated with AREG. (C): The number of actin stress fiber in each granulosa cell cultured on serum-coated well or free well with or without AREG. Values are the mean ± SEM of fifteen granulosa cells. *; Significant differences were observed compared with the control (p<0.05).

When granulosa cells were cultured on uncoated wells, the morphology of granulosa cells was circular and the cell membrane was lined with actin polymers (Fig 4A). The addition of AREG did not change the shape of granulosa cells, and did not increase the size of granulosa cells (Fig 4A and 4B). Stretching of granulosa cells was observed when they were cultured on the serum-coated glass plate (Fig 4A and 4B). The cell membrane was lined with actin polymers and spots of actin polymers were also observed in the cytoplasm of granulosa cells cultured on the serum-coated plate (Fig 4A). A larger increase in the surface area of granulosa cells was induced by addition of AREG when granulosa cells were cultured on the serum-coated plate (Fig 4B). In granulosa cells treated with AREG on serum-coated plate, actin stress fibers in the cytoplasm had formed, and the number of fibers was significantly higher than in granulosa cells cultured on the serum-coated plate but without AREG treatment (Fig 4C).

**Fig 5:**
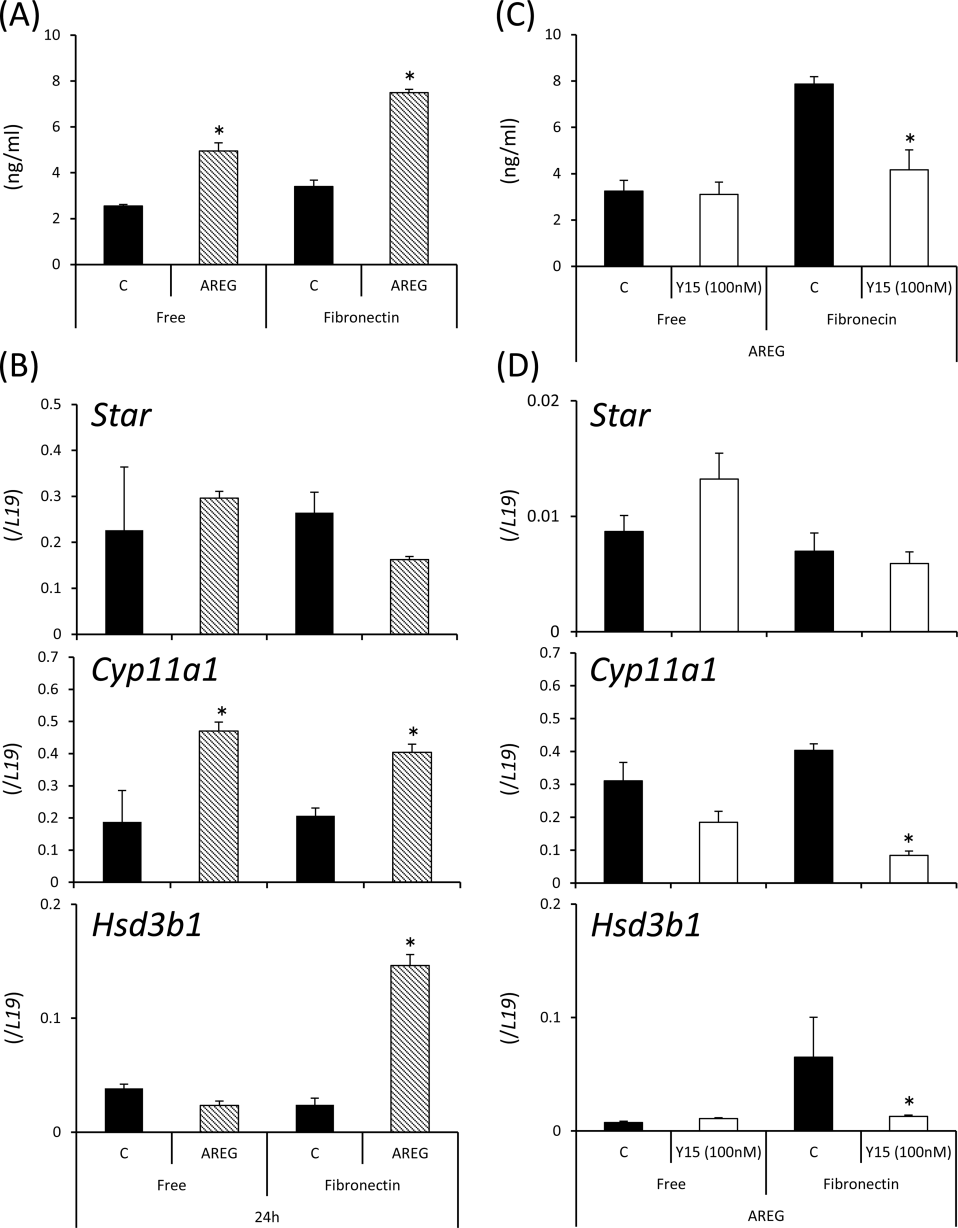
Roles of AREG and fibronectin in progesterone production of granulosa cells. (A): Progesterone concentrations in culture supernatants of granulosa cells treated for 24 h with or without AREG on uncoated or fibronectin-coated wells. Values are the mean ± SEM of three replicates. Data were statistically analyzed by two-way ANOVA. Because the significant difference was observed for each factor, the comparison analysis was performed by the Student’s t-test. *; AREG significantly increased the area of granulosa cells cultured on fibronectin-coated wells or free wells (p<0.05). Free: granulosa cells cultured on uncoated wells; Fibronectin: granulosa cells cultured on fibronectin-coated wells; C (control): granulosa cells treated without AREG; AREG: granulosa cells treated with AREG. (B): Expression of genes involved in progesterone production (*Star*, *Cyp11a1*, and *Hsd3b1*) in granulosa cells treated for 24 h with or without AREG on uncoated or fibronectin-coated wells. Values are the mean ± SEM of three replicates. Data were statistically analyzed by two-way ANOVA. Because the significant difference was observed for each factor, the comparison analysis was performed by the Student’s t-test. *; AREG significantly increased the area of granulosa cells cultured on fibronectin-coated wells or free wells (p<0.05). **AREG significantly increased the area of granulosa cells cultured on fibronectin-coated wells (p<0.05). Free: granulosa cells cultured on uncoated wells; Fibronectin: granulosa cells cultured on fibronectin-coated wells; C (control): granulosa cells treated without AREG; AREG: granulosa cells treated with AREG. (C): Additional effects of Y15 on AREG-induced progesterone production in granulosa cells cultured on fibronectin-coated wells or free-wells. Values are the mean ± SEM of three replicates. *; Y15 treatment significantly decreased the progesterone level when granulosa cells were cultured on fibronectin-coated wells (p<0.05). (D): The treatment with Y15 inhibited AREG-induced expression of genes involved in progesterone production (*Star*, *Cyp11a1*, and *Hsd3b1*) in granulosa cells. Values are the mean ± SEM of three replicates. *; Y15 treatment significantly inhibited the gene expression levels compared with each control (p<0.05).

The addition of AREG significantly increased the level of progesterone in the culture supernatant with induction of *Cyp11a1* expression in granulosa cells cultured on both uncoated and fibronectin-coated plates. The fibronectin-coated plate induced significantly higher progesterone production in AREG-stimulated granulosa cells (Fig 5A). The expression of *Hsd3b1* was only induced by AREG when granulosa cells were cultured on fibronectin-coated plate (Fig 5B). Treatment with Y15 did not significantly affect the level of progesterone when granulosa cells were cultured without the fibronectin-coating. However, the high level of progesterone secreted from granulosa cells treated with AREG on fibronectin-coated wells was significantly decreased by the additional Y15 with reductions in *Cyp11a1* and *Hsd3b1* expression (Fig 5C and 5D).

### Binding of fibronectin to integrins and FAK activity induce cumulus expansion and oocyte maturation of COCs

**Fig 6:**
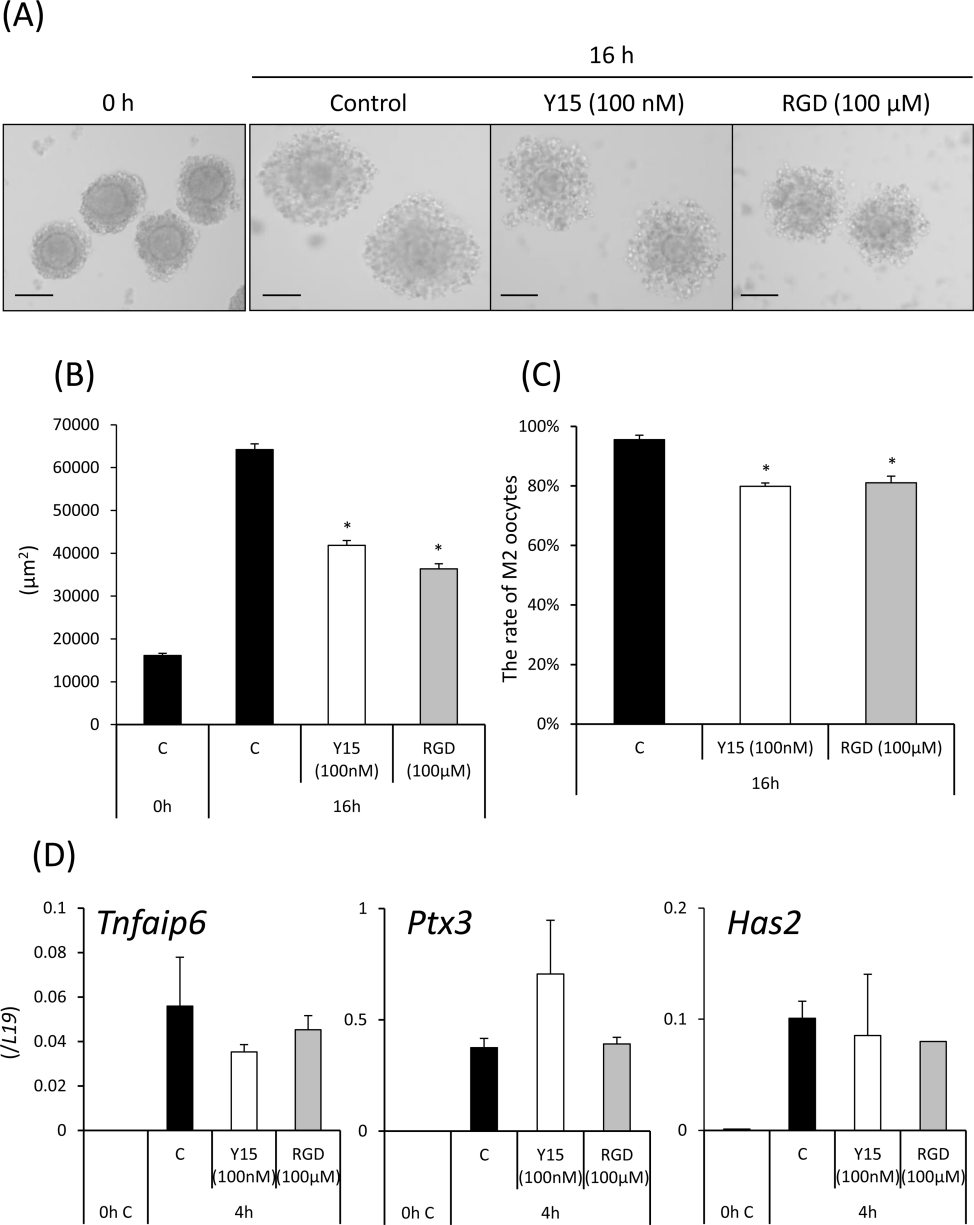
Effects of FAK inhibitor Y15 and RGD peptide on cumulus expansion *in vitro*. (A,B): The surface area of expanded COCs was measured using the BZ-9000 microscope. COCs were isolated from ovaries of mice primed with eCG for 48 h. Non-expanded COCs were selected, and 50 COCs were cultured in a 50 μl drop of defined medium containing 1% FBS and 100 ng/ml AREG. Some COCs were treated with AREG and 100 nM Y15 or 100 μM RGD peptide (RGD; arginine-glycine-aspartate, tripeptide). After 16 h, the area of each expanded COC was measured using the BZ-9000 microscope. Scale bar is 100 μm. The left panel shows an image of an expanded COC in each treatment group after culture. Values are the mean ± SEM of fifteen COCs each in three replicates. *; Y15 or RGD peptide significantly suppressed cumulus expansion compared with the control (p<0.05). (C): The rate of metaphase II stage oocytes when COCs were cultured with Y15 or RGD peptide. *; Y15 or RGD peptide significantly decreased the rate of metaphase II (M2) oocytes compared with the control (p<0.05). (D): Expression of genes involved in cumulus expansion (*Has2*, *Tnfaip6*, and *Ptx3*). Each value is presented as the gene expression level. Values are the mean ± SEM of three replicates.

Cumulus expansion was induced *in vitro* by treatment with AREG, and the induction was suppressed by either Y15 or RGD peptide (Fig 6A and 6B). Progression to the metaphase II stage was significantly inhibited by treatment with either Y15 or RGD peptide when COCs were cultured in FBS-containing medium (Fig 6C). In cumulus cells of COCs treated with Y15 or RGD peptide, AREG-induced expression of genes related to cumulus expansion (*Has2*, *Tnfaip6*, and *Ptx3*) was also induced to the same levels in COCs without any inhibitors (Fig 6D).

## Discussion

FAK is one of key regulators of cellular functions due to remodeling of the cytoskeleton; however, there is little information in ovary during ovulation process. The present study revealed that in ovary, FAK was expressed and was transiently phosphorylated during ovulation process. The phosphorylation of FAK was dependent on EGFR ligand AREG and fibronectin in cultured granulosa cells. The physiological roles of FAK in periovulatory follicles were examined using a pharmacological approach by co-injecting FAK inhibitor Y15 into super ovulation-induced mice. Y15 directly binds to Tyr397 of FAK to reduce its kinase activity, which markedly suppresses the migration of cancer cells [23]. During ovulation process, detachment and migration of cumulus cells are induced to create space between cumulus cells for accumulation of the hyaluronan-rich matrix [6]. The hCG-induced expansion of cumulus cell layers was suppressed by treatment with Y15 concomitantly with the reductions of *Has2* and *Ptgs2* expression. *In vitro* culture also revealed that both Y15 and RGD peptide directly but slightly suppressed cumulus expansion without any expression of genes involved in hyaluronan synthesis or its accumulation. The differences would be explained by AREG- and PGE2-positive feedback loop to induce the expansion of cumulus cell layer during ovulation process [3]. FAK was required for the production of PGE2 and AREG in granulosa cells. In cumulus cells, the PGE2 and AREG induced *Has2* expression and FAK was involved in the migration of cumulus cells but not the production of hyaluronic acid during ovulation process. In some knockout (KO) mouse models with defects in cumulus expansion, such as *Ptgs2*KO, *Ptx3*KO and *Tnfaip6*KO mice, the number of ovulated oocytes is also significantly but not completely decreased [24][25][26][27]. In the present study, treatment with Y15 significantly decreased the number of ovulated oocytes, and several antral follicles but less numbers of corpus luteum were observed in the treated ovary. These data indicate that FAK in both granulosa cells and cumulus cells indirectly or directly induces the migration of cumulus cells, which is required for successful ovulation process.

In glomerular podocytes, Y15 also affects the cell shape [28], indicating that FAK is involved in cellular functions due to remodeling of the cytoskeleton because the change in cell shape is driven by actin depolymerization or polymerization [29]. Formation of the corpus luteum, including changes of the granulosa cell shape, was also suppressed by injection of Y15 with hCG. The circular shape of granulosa cells in preovulatory follicles was stretched with stress fiber formation. Stress fibers consist of actin and myosin, and actin polymerization is induced in a Rho GTPase family-dependent manner [30][31][32]. FAK is also involved in the formation of stress fibers, because FAK activity increases in Rho-stimulated cells [33][34][35]. In this study, treatments with both AREG and fibronectin, but not either alone induced the phosphorylation of Tyr925 in FAK in granulosa cells with the formation of stress fibers. The inductions were correlated with the expression of genes involved in granulosa cell luteinization. In epithelial cells, mechanical tension during wound closure also induces stress fiber assemblies, which then differentiate into myoepithelial cells [36]. Mesenchymal stem cells (MSCs) induced to undergo chondrogenic differentiation have more pronounced actin stress fibers compared with undifferentiated MSCs [37]. Moreover, actin stress fiber formation in response to TGF-β is also important for myofibroblast differentiation [38]. Thus, AREG and fibronectin synergistically progress the luteinization of granulosa cells via FAK-induced stress fiber formation.

Interestingly, the addition of Y15 or RGD peptide to oocyte maturation medium decreased the rate of the MII stage when COCs were cultured with AREG in the presence of FBS. In the *in vitro* maturation (IVM) of oocytes, serum-free chemically defined medium results in a much lower maturation rate compared with serum-containing medium [39]. Removal of cumulus cells before IVM also dramatically decreases the rate of maturation in human, porcine, cattle and mouse oocytes [40][41][42]. Because serum contains fibronectin and cumulus cells supply fibronectin, the low maturation rate of denuded oocytes or in chemically defined medium may be induced by the lack of fibronectin during oocyte maturation process. In the fields of human infertility care and regenerative medicine, chemical defined medium is highly recommended to reduce the risk of infection [43]. Moreover, removal of cumulus cells is required to check the maturation of oocytes recovered from hormone-stimulated patients before injection of sperm (ICSI). Thus, it is possibility that the addition of fibronectin is to improve the IVM technique of human oocytes.

In conclusion, during ovulation, the structure of follicles changes dramatically with the alteration of microenvironments in periovulatory follicles. The presence of fibronectin in granulosa cell layers and the space between cumulus cells create the basis for their differentiation. EGF-like factor acts on granulosa cells and cumulus cells enclosed by fibronectin to drive the dynamic changes of the cytoskeleton with the differentiation of each cell type, cumulus expansion of cumulus cells, and luteinization of granulosa cells. Thus, it is concluded that both EGF-like factor and fibronectin synergistically act on granulosa and cumulus cells to induce successful ovulation process.

## Supporting information

S1 Fig The effect of FAK inhibitor Y15 in granulosa cells during ovulation process. (A): FAK inhibitor Y15 was injected into three immature mice with hCG and the ovary was collected at 4 h after hCG injection. The effects of Y15 on the phosphorylation status of pFAK (Tyr397) and pFAK (Tyr925) were detected by western blotting.
(B): FAK inhibitor Y15 (100 nM) was added in cultured granulosa cells for 4 h with AREG on serum-coated wells. The effects of Y15 on the phosphorylation status of pFAK (Tyr397) and pFAK (Tyr925) were detected by western blotting. β-actin was used as a loading control. The intensity of the bands was analyzed using a Gel-Pro Analyzer. Values are mean +/- SEM of 3 replicates.
(PDF)

S2 Fig The protein expression of fibronectin and integrin in the mouse ovary during ovulation. Expression of fibronectin, integrin β1, and β-actin in whole ovary samples was detected by western blot analyses. The ovary was collected from mice treated with hCG for 0, 4, 8, or 16 h at 48 h after eCG injection. β-actin was used as a loading control. The intensity of the bands was analyzed using a Gel-Pro Analyzer. Values are mean +/- SEM of 3 replicates.
(PDF)

S3 Fig The negative control sections for immunofluorescence staining in Fig 1. The ovarian section was treated with only Cy3- or FITC-labeled secondary antibody. The nucleus was counterstained with DAPI. Scale bar is 100 μm.
(PDF)

S4 Fig Immunofluorescence staining of mature oocyte treated with Y15. COCs were isolated from preovulatory follicles at 48 h after eCG injection. Non-expanded COCs were selected and were cultured in the medium containing 1% (v/v) of FBS with 100 ng/ml AREG and/or 100 nM FAK inhibitor (Y15) in the presence of 4 mM of hypoxanthine for 16 h. Red signal is F-actin, green signal is α/β Tubulin and blue signal is DAPI.
(PDF)

S1 Table Primer list. (DOCX)

S2 Table Antibody list. (DOCX)
