# Supplementary figures and images for: Inductions of granulosa cell luteinization and cumulus expansion are dependent on the fibronectin-integrin pathway during ovulation process in mice
Source: PLoS One. 2018 Feb 8;13(2):e0192458. doi: 10.1371/journal.pone.0192458 (PMC5805282; doi:10.1371/journal.pone.0192458)

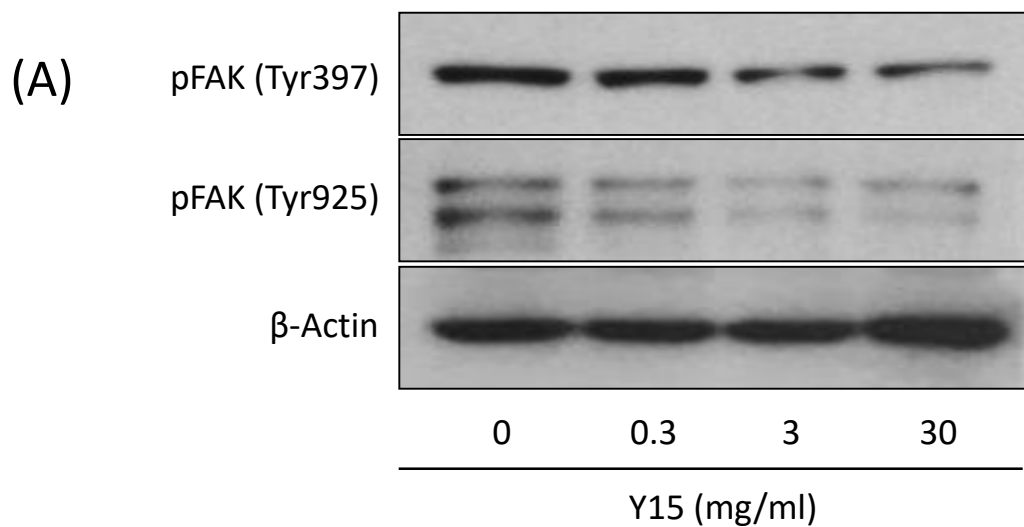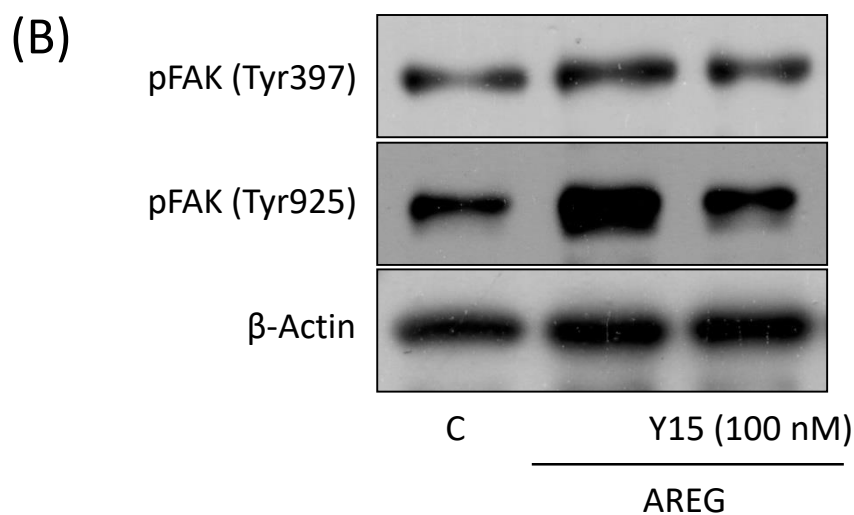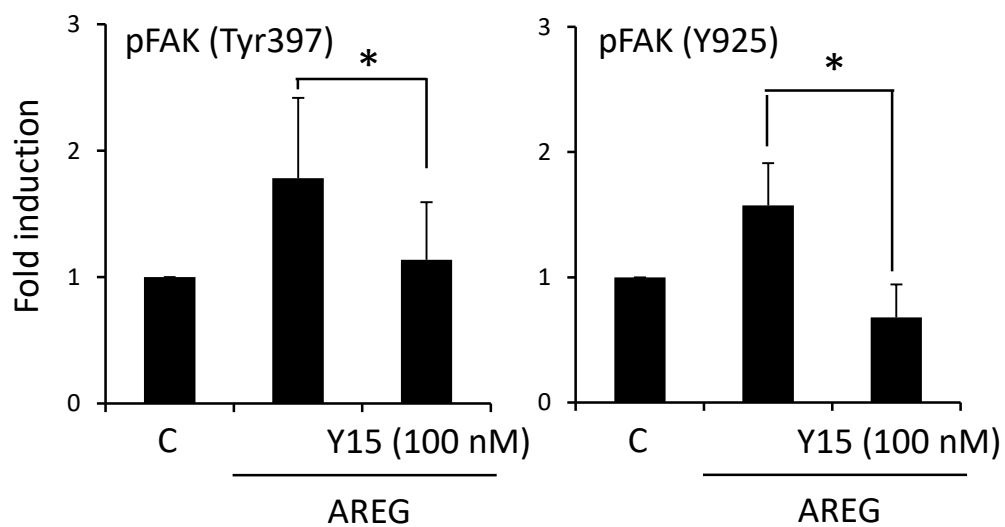

Supplement: S1 Fig — (A): FAK inhibitor Y15 was injected into three immature mice with hCG and the ovary was collected at 4 h after hCG injection. The effects of Y15 on the phosphorylation status of pFAK (Tyr397) and pFAK (Tyr925) were detected by western blotting. (B): FAK inhibitor Y15 (100 nM) was added in cultured granulosa cells for 4 h with AREG on serum-coated wells. The effects of Y15 on the phosphorylation status of pFAK (Tyr397) and pFAK (Tyr925) were detected by western blotting. β-actin was used as a loading control. The intensity of the bands was analyzed using a Gel-Pro Analyzer. Values are mean +/- SEM of 3 replicates. (PDF) [file pone.0192458.s001.pdf]

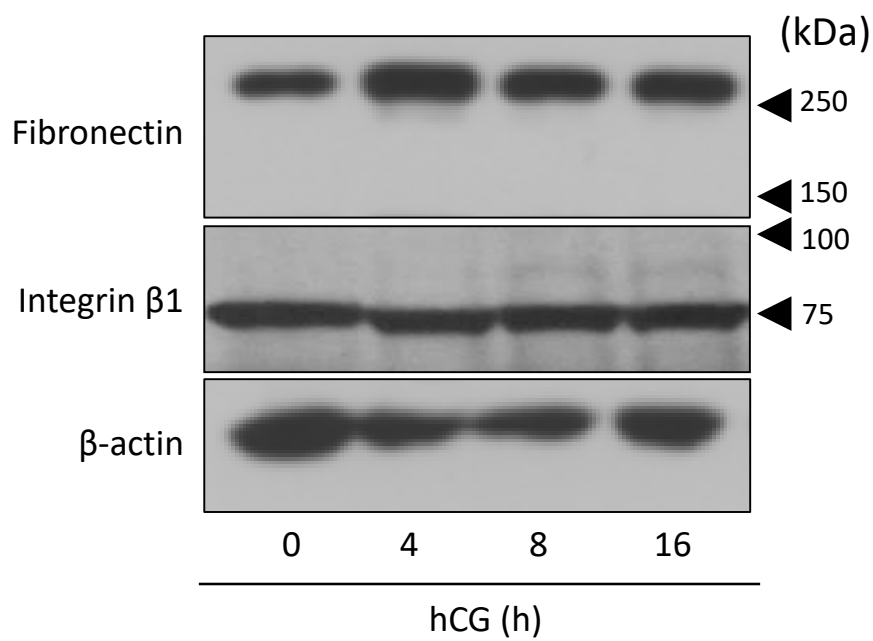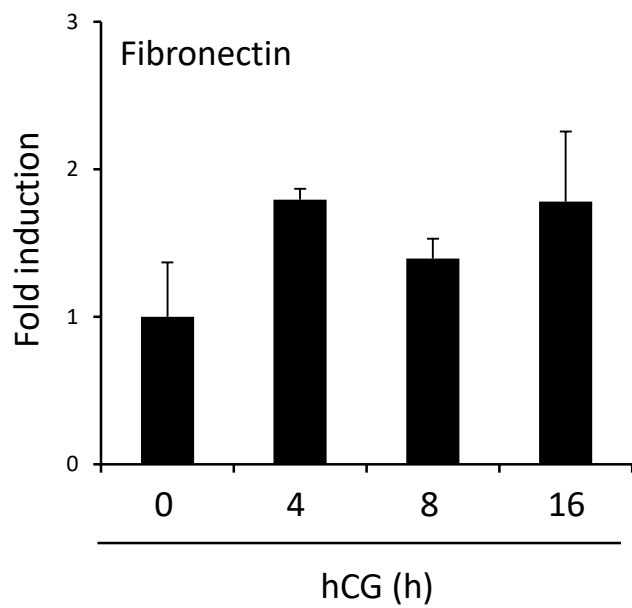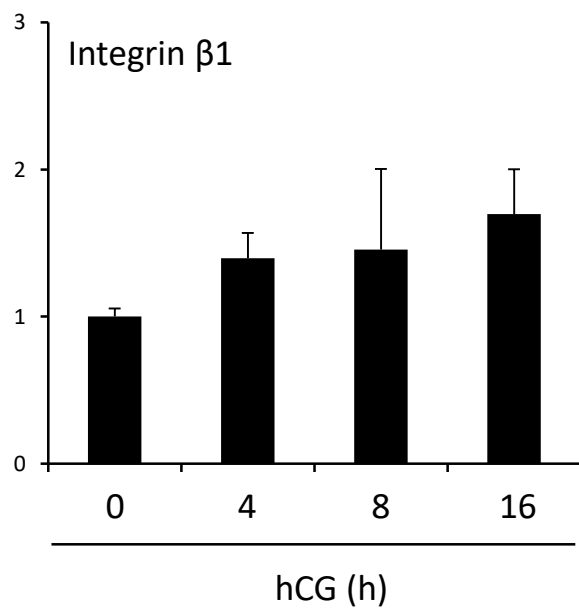

Supplement: S2 Fig — Expression of fibronectin, integrin β1, and β-actin in whole ovary samples was detected by western blot analyses. The ovary was collected from mice treated with hCG for 0, 4, 8, or 16 h at 48 h after eCG injection. β-actin was used as a loading control. The intensity of the bands was analyzed using a Gel-Pro Analyzer. Values are mean +/- SEM of 3 replicates. (PDF) [file pone.0192458.s002.pdf]

Control (normal MII stage)

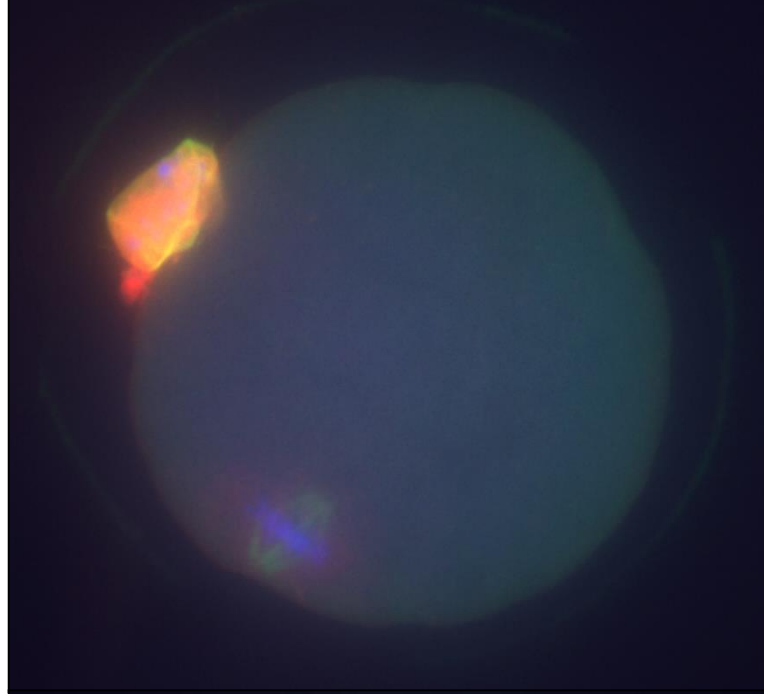

Y15 (100 nM) (abnormal MII stage)

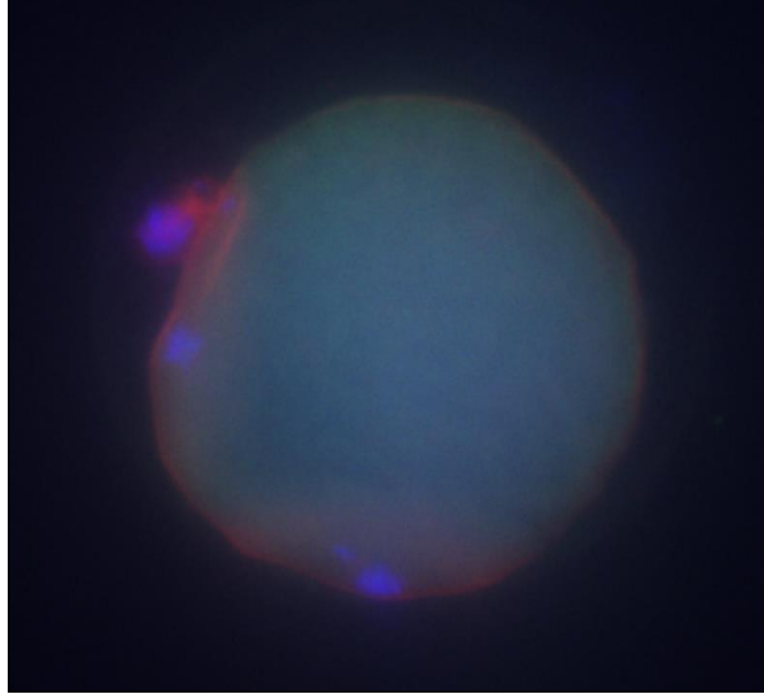

16h

Supplement: S4 Fig — COCs were isolated from preovulatory follicles at 48 h after eCG injection. Non-expanded COCs were selected and were cultured in the medium containing 1% (v/v) of FBS with 100 ng/ml AREG and/or 100 nM FAK inhibitor (Y15) in the presence of 4 mM of hypoxanthine for 16 h. Red signal is F-actin, green signal is α/β Tubulin and blue signal is DAPI. (PDF) [file pone.0192458.s004.pdf]
