# Supplementary material for: Inductions of granulosa cell luteinization and cumulus expansion are dependent on the fibronectin-integrin pathway during ovulation process in mice
Source: PLoS One. 2018 Feb 8;13(2):e0192458. doi: 10.1371/journal.pone.0192458 (PMC5805282; doi:10.1371/journal.pone.0192458)
Supplement: S3 Fig — The ovarian section was treated with only Cy3- or FITC-labeled secondary antibody. The nucleus was counterstained with DAPI. Scale bar is 100 μm. (PDF) [file pone.0192458.s003.pdf]

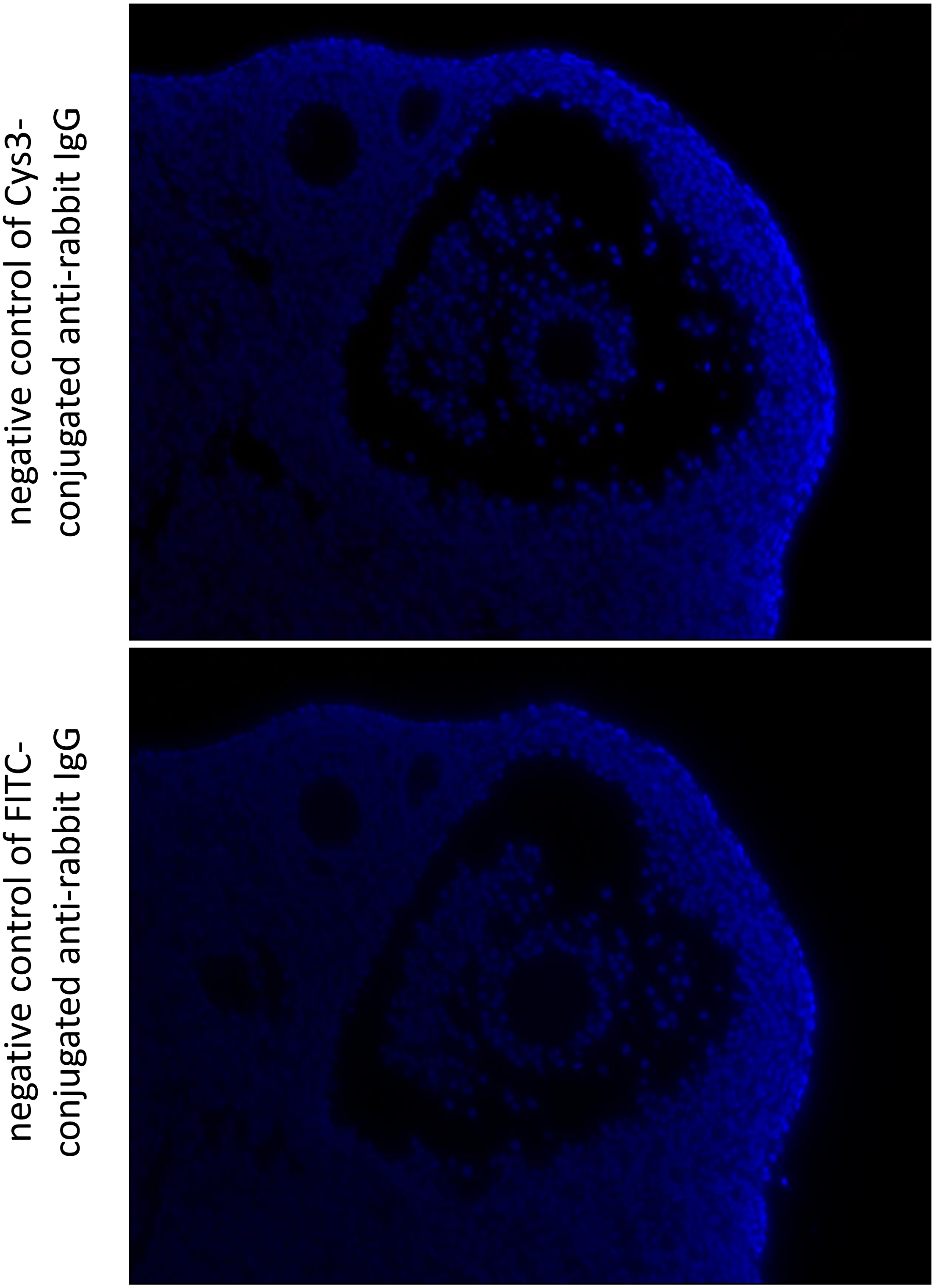
negative control of Cys3-conjugated anti-rabbit IgG
negative control of FITC-conjugated anti-rabbit IgG

hCG 8h
