# Supplementary material for: Inductions of granulosa cell luteinization and cumulus expansion are dependent on the fibronectin-integrin pathway during ovulation process in mice
Source: PLoS One. 2018 Feb 8;13(2):e0192458. doi: 10.1371/journal.pone.0192458 (PMC5805282; doi:10.1371/journal.pone.0192458)
Supplement: S1 Table — (DOCX) [file pone.0192458.s005.docx]

| Gene | Forward Primer | Reverse Primer | Size (bp) | Anneling temperature |
| --- | --- | --- | --- | --- |
| *Star*  *Cyp11a1* | 5’-GCAGCAGGCAACCTGGTG-3’  5’-GGGAGACATGGCCAAGATGG-3’ | 5’-TGATTGTCTTCGGCAGCC-3’  5’-CAGCCAAAGCCCAAGTACCG-3’ | 247  279 | 60  60 |
| *Hsd3b1* | 5’-TGGGGAGAGAAGTCCATTCA-3’ | 5’-GGAGCCCCCATTCCTTACTA-3’ | 196 | 60 |
| *Has2* | 5’-AGACATTCAATGGGGGTTGG-3’ | 5’-CCACACAAAGCATGGCAAGT-3’ | 191 | 60 |
| *Tnfaip6* | 5’-GTCTGTGCTGCTGGGTGGAT-3’ | 5’-CGTACTTGAGCCGGATGTGC-3’ | 268 | 60 |
| *Px3* | 5’-TGGCTGAGACCTCGGATGAC-3’ | 5’-GCGAGTTCTCCAGCATGATGA-3’ | 153 | 60 |
| *L19* | 5’-GGCATAGGGAAGAGGAAGG-3’ | 5’-GGATGTGCTCCATGAGGATGC-3’ | 199 | 60 |

S1 Table. Primer list
