# Supplementary material for: Inductions of granulosa cell luteinization and cumulus expansion are dependent on the fibronectin-integrin pathway during ovulation process in mice
Source: PLoS One. 2018 Feb 8;13(2):e0192458. doi: 10.1371/journal.pone.0192458 (PMC5805282; doi:10.1371/journal.pone.0192458)
Supplement: S2 Table — (DOCX) [file pone.0192458.s006.docx]

| Target | The name of antibody | Catalog No. | Species |
| --- | --- | --- | --- |
| Fibronectin  Integlin β1 | Anti-FN1 Polyclonal Antibody  Anti-ITGB1 Antibody | ORIGENE (AP06602PU-N)  LSBio (LS-C202006) | Rabbit  Mouse |
| Integlin α5 | Anti-ITGA5 Polyclonal Antibody | Bioss (bs-0567R-FITC) | Rabbit |
| FAK | Anti-FAK monoclonal antibody (H-1) | Santa Cruz (SC-1688) | Mouse |
| pFAK (Y397) | Anti-pFAK (Y397) Antibody | Cell signaling (3283S) | Rabbit |
| pFAK (Y925) | Anti-pFAK (Y925) Antibody | Cell signaling (3284S) | Rabbit |
| β-actin | Anti-β-actin monoclonal antibody | Sigma (A5316) | Mouse |
| α/β-Tubulin | Anri-α/β-Tubulin Antibody | Cell signaling (2148S) | Rabbit |
| F-Actin | Rhodamine phalloidin | Invitrogen (R415) |  |

Table S2. Antibody list
